# A nutrition and conditioning intervention for natural bodybuilding contest preparation: observations and suggestions

**DOI:** 10.1186/s12970-015-0111-x

**Published:** 2015-12-21

**Authors:** Paulo Gentil

**Affiliations:** College of Physical Education and Dance, Federal University of Goias, Avenida Esperança s/n, Campus Samambaia, Goiania, 74.690-900 GO Brazil

**Keywords:** Bodybuilder, Resistance training, High intensity interval training, Protein ingestion, Carbohydrate ingestion

## Abstract

Bodybuilding is full of myths and practices that are contrary to the scientific literature, which can lead to health problems. Adopting a scientifically designed approach is very important, as it may help bodybuilders to achieve better results while preserving their health. However, I have some criticism regarding some practices adopted in the referred article as *ad libitum* ingestion of sugar-free cordial and flavored tea and the performance of the exercise in fasted state, as it seems to bring no benefit and have some potential problems. Some suggestion are made in order to preserve FFM, like changing training split and exercise selection; increasing carbohydrate ingestion and decreasing protein intake; changing the resistance training stimuli and reducing the volume of aerobic exercises and increase its intensity.

I would like to congratulate the authors for the interesting case study [1]. Bodybuilding is full of myths and practices that are contrary to the scientific literature, which can lead to health problems, as previously reported in this journal [2]. Adopting a scientifically designed approach is very important, as it may help bodybuilders to achieve better results while preserving their health.

However, I have some criticism regarding the adopted *ad libitum* ingestion of sugar-free cordial and flavored tea. Although sweeteners may be valuable in substitution to sugar or high caloric foods [3], some large scale prospective cohort studies found positive correlation between artificial sweetener ingestion and weight gain [4, 5]. There is a suggestion that artificial sweeteners do not activate the food reward pathways and may encourage sugar dependence [5], which may be deleterious to diet adherence in long term. Moreover, the regular use of sweetener has been linked to glucose intolerance, as suggested by recent reviews [6, 7]. So, it is recommended to employ a systematic reduction of sugar ingestion, without any substitution for sweeteners [5].

Another questionable practice is the performance of the exercise in fasted state, as it brings no benefit in terms of fat loss [8] and can negatively impact energy expenditure and fat metabolism [9]. So, it is advisable to progressively change this habit.

That said, I would like to make some observations and suggestions. The 2014 Olympia Men’s Physique champion was 173 cm with 88 kg during off season and 75 kg during contest. It results in a BMI of 30 and 25, respectively. The participant of the study had a BMI of 27 and dropped to 23 after the preparation. It seems prudent to adopt strategies to preserve FFM loss, since he is probably undersized for competing at high level.

It is important to note that the volume of training used in this study is considerably high and previous studies suggested that it might be necessary to reduce resistance training volume during caloric restriction [10]. One first thought is to change training split and exercise selection to avoid excessive stress. Previous studies suggest that multi joint exercises promote sufficient stimuli for gaining muscle size and strength in all muscles involved in the exercise [11] and there seems to be no additional benefit in performing single joint exercises [12, 13]. Therefore the use of separate sessions to train the arms does not seem to be necessary and may lead to an unbalance between stress and recovery.

The proposed diet decreased carbohydrate ingestion to 23–25 % of the calories ingested and established protein intake at approximately 2.4 g per kg of body weight. Although the benefits of high protein diets on weight loss are highlighted [14], the results of meta-analyses indicate that the quantity of protein necessary to promote weight management and preserve lean mass lies somewhere between 1.2 and 1.6 g/kg [15–17]. The highest suggestion of protein ingestion was published by Phillips & Van Loon [18], who recommended to increase protein intake to 1.8 to 2.7 g/kg in order to optimize the ratio of fat-to-lean tissue mass loss during hypoenergetic periods. However, the authors showed no convincing evidence for proposing this number.

Additionally, a systematic review by Helms et al. [19] suggested that 2.3-3.1 g/kg FFM to be appropriate for lean, resistance-trained athletes in hypoenergetic conditions. However it is important to note that, among the studies used by Helms et al, only two used protein intakes over 2 g/kg [20, 21] and, among them, only one compared the effects of different protein intakes [21]. In this study, by Mettler et al. [21], the low protein group ingested only 1 g/kg of protein, which is very low. Therefore, although increasing protein intake may be a valuable to strategy as suggested by recent studies [22], it is important for coaches and dietitians to know that there are other nutritional strategies that can be used during precontest, especially if their athletes do not tolerate severe restrictions in carbohydrate or fat. It is important to note that the literature recommends the ingestion of 1.2 to 2 g of protein per kg of body weight for strength athletes [23–25] and there are some suggestions of no benefit on increasing it above this level [26].

As low carbohydrate ingestion may compromise exercise performance [18], one suggestion would be to increase carbohydrate ingestion and decrease protein intake. Another suggestion is to change the resistance training stimuli. Although it is commonly suggested that the optimal resistance training protocol for promoting fat loss should be done with high volume and high repetitions, there is evidence that low volume and high intensity workouts may promote more favorable acute [27] and chronic outcomes [28, 29] for those wanting to lose fat. Moreover, if one is to adopt low carbohydrate diets, using low repetition and high load resistance training protocols may be advantageous, since this type of training rely less on the glycolitic system [30].

Finally, it would be recommended to reduce the volume of aerobic exercises and increase its intensity, since it has been shown that the higher the volume of aerobic exercise, the lower the muscle hypertrophy [31], in this regard it is important to note that running may have a more negative effect than cycling [32]. On the other hand, the higher the exercise intensity, the higher the fat loss [31] and it seems that the effect of regular aerobic exercise on body fat is negligible [32]. Taken together, this make high intensity interval training more recommended than long duration and low intensity training for both losing fat and preserving fat free mass.
